# Lethal Mutants and Truncated Selection Together Solve a Paradox of the Origin of Life

**DOI:** 10.1371/journal.pone.0021904

**Published:** 2011-07-26

**Authors:** David B. Saakian, Christof K. Biebricher, Chin-Kun Hu

**Affiliations:** 1 Institute of Physics, Academia Sinica, Nankang, Taipei, Taiwan; 2 Yerevan Physics Institute, Yerevan, Armenia; 3 National Center for Theoretical Sciences: Physics Division, National Taiwan University, Taipei, Taiwan; 4 Max-Planck-Institute for Biophysical Chemistry, Göttingen, Germany; 5 Department of Physics, Beijing Normal University, Beijing, China; City of Hope National Medical Center and Beckman Research Institute, United States of America

## Abstract

**Background:**

Many attempts have been made to describe the origin of life, one of which is Eigen's cycle of autocatalytic reactions [Eigen M (1971) Naturwissenschaften 58, 465–523], in which primordial life molecules are replicated with limited accuracy through autocatalytic reactions. For successful evolution, the information carrier (either RNA or DNA or their precursor) must be transmitted to the next generation with a minimal number of misprints. In Eigen's theory, the maximum chain length that could be maintained is restricted to 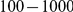 nucleotides, while for the most primitive genome the length is around 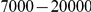. This is the famous error catastrophe paradox. How to solve this puzzle is an interesting and important problem in the theory of the origin of life.

**Methodology/Principal Findings:**

We use methods of statistical physics to solve this paradox by carefully analyzing the implications of neutral and lethal mutants, and truncated selection (i.e., when fitness is zero after a certain Hamming distance from the master sequence) for the critical chain length. While neutral mutants play an important role in evolution, they do not provide a solution to the paradox. We have found that lethal mutants and truncated selection together can solve the error catastrophe paradox. There is a principal difference between prebiotic molecule self-replication and proto-cell self-replication stages in the origin of life.

**Conclusions/Significance:**

We have applied methods of statistical physics to make an important breakthrough in the molecular theory of the origin of life. Our results will inspire further studies on the molecular theory of the origin of life and biological evolution.

## Introduction

The puzzle about the origin of life has attracted the attention of curious minds from the dawn of human civilization. Since the development of molecular biology, it has been known that the information carriers of living organisms, from humans to bacteria and viruses, are DNA and RNA. An essential step in solving the puzzle about the origin of life at a molecular level is to understand the replication and evolution of information carriers. For this purpose, Eigen [1], [2] proposed a cycle of autocatalytic reactions.

Primordial life molecules are replicated through autocatalytic reactions with a limited accuracy, i.e. an error rate in the order of 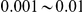 [3], [4]. For successful evolution, genetic information must be transmitted to the next generation with a minimal number of misprints. With an error rate in the order of 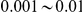 [3], [4], the maximum length of information carrier that could be maintained is estimated in Eigen's theory [1], [2] to be 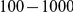. For the most primitive genome, the length is estimated by Gil *et al* [5] to be around 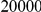 nucleotides, and by Kun *et al* [6] to be around 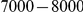 nucleotides. The former includes the core bacterial gene set, and the latter includes only the key information carrier. The big gap between 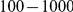 and 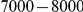 is the famous error catastrophe paradox.

In this paper, we use methods of statistical physics to solve the error catastrophe paradox by carefully analyzing the roles of neutral networks, in which each mutant in the network has about the same reproduction rate as the master sequence [6], [7], lethal mutants, i.e., mutants with a zero reproduction rate [8], [9], and truncated selection, i.e., when the mutants with Hamming distances from the master sequence larger than a critical value 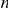 have a zero reproduction rate [10]–[12]. We calculate the impact of neutrality for the neutral thick hierarchic tree, and derive simple exact formulae for the case of the neutral network-like fitness landscape. The importance of neutral network-like fitness landscapes is widely known [13]–[15].

The neutrality phenomenon with perfect (the neutral mutants have exactly the same fitness as the master sequence) and extensive neutrality has been considered in [6], [16], [17], and a large increase in the mean fitness due to neutrality has been found. However, mutants with both perfect and extensive neutrality are not realistic and cannot be found in real biological systems. A more realistic case is imperfect extensive neutrality, to be discussed below.

In the present paper, we consider different versions of neutrality: the neutral network-like fitness landscape, which is very popular among biologists, and the more involved thick hierarchic tree landscape, to be defined later. In both cases, we derive analytical results for the mean fitness and for the probabilities of the main sequences appearing. Our result for the mean fitness of the neutral network is consistent with the rigorous result of Nimwegen *et al* [14]. In all cases we consider, the modification of the mean fitness due to neutrality involves a small factor of the order 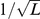. The corresponding change in the critical chain length is negligible in solving the error catastrophe paradox. We will discuss the extensive neutrality [6], [16], [17] in the subsection **Extensive neutrality**, below. According to our analysis of the experimental data, the increase in mean fitness is also negligible for the observed case of imperfect extensive neutrality.

Applying statistical physical methods used in earlier papers [18]–[28] to the Eigen model with lethal mutants and truncated selection, we analyze the paradox of the origin of life. We find that the combined action of lethal mutants and truncated selection makes the error threshold reach the required genome length for the origin of life and thus solves the paradox of the origin of life.

Here we use the concept from the statistical physics of spin models [29] to review briefly Eigen's theory of the cycle of autocatalytic reactions [1], [2].

The genetic information of a biological system is stored in the DNA or RNA sequence. Eigen used models similar to the one-dimensional Ising model [29] with 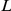 spins to represent DNA or RNA of 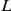 bases, and considered the time evolution of the probability distribution 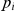, 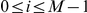, of 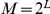 spin configurations 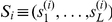 corresponding to 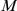 DNA or RNA sequences, with 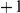 "spin" representing purines (R) and 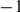 "spin" representing pyrimidines (Y) in a sequence. Every sequence 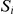 is assigned a value of the fitness function, 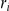. The number 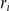 represents the reproduction rate of 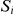.

**Figure 1:**
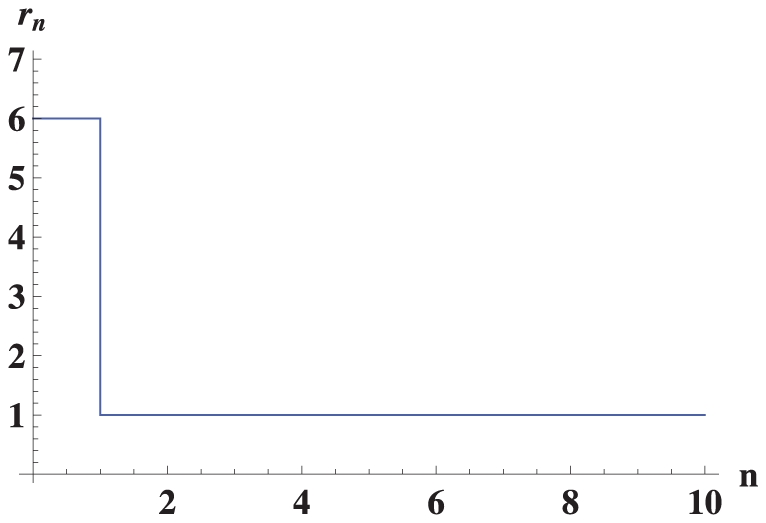
Fitness 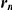 versus Hamming distance 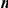 from the peak sequence for the single-peak fitness landscape.

**Figure 2:**
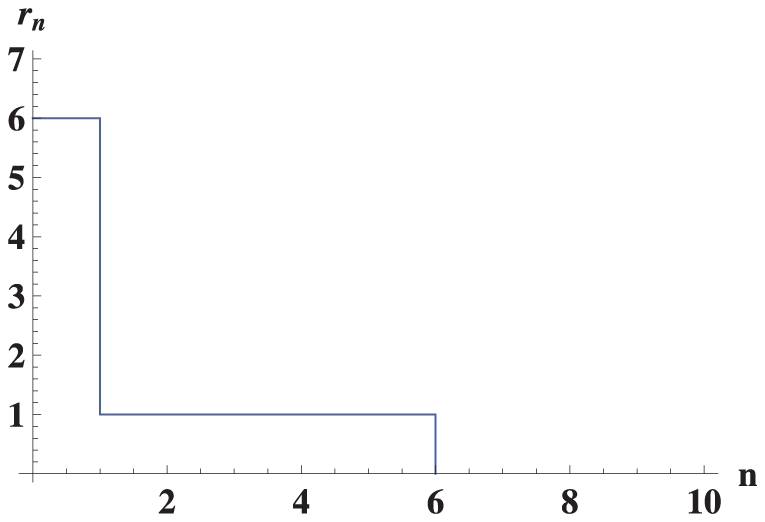
Fitness 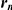 versus Hamming distance 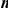 from the peak sequence for the truncated fitness landscape.

In the simplest case of the single-peak fitness function, there is only one peak configuration or master sequence, say 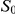, which has the largest value of fitness function so that 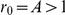, and 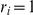 for 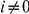, as shown in Fig. 1. Configuration 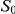 can be chosen to be 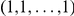, i.e., all spins take 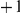, without the loss of generality. The 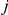-th sequence 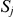 can change into the 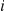-th sequence 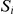 via mutation. The Hamming distance between configurations 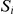 and 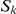, i.e., the number of minimal mutation flips from 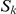 to 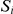, is denoted by 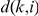. In the truncated selection, the fitness function is zero after some Hamming distance from the master sequence. A typical example is shown in Fig. 2.

In Eigen's theory [1], [2], an information carrier reproduces at a certain rate 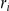, producing offspring of the parental type with the probability 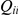 and offspring of the mutant type 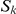 (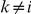) with the probability 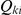. The probabilities 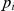 for different types (sequences) 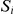, 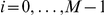, satisfy the set of equations
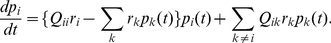
(1)Here, 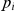 satisfy the normalization condition 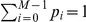; the elements of the mutation matrix are 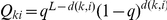; 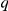 is the probability of errorless replication per nucleotide. The diagonal terms of the mutation matrix are 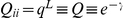, where 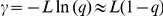 is the parameter of mutation in the Eigen model. Two sequences 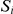 and 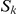 are neighbors if and only if 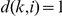. For 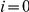, the second term on the right-hand side of Eq. (1) represents the back mutation from mutants to the master sequence.

For the single peak fitness landscape with 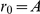 and 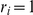, for 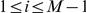 (Fig. 1), Eigen derived the following restriction for the length of genome [1], [2]

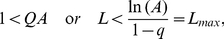
(2)for 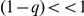, where 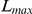 is the maximal allowed genome length. If we neglect with a 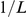 accuracy a small contribution from the second term in the right-hand side of Eq. (1), i.e., from back mutations, we can easily show that the steady state probability 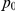 for the master sequence and the mean fitness 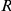 are given by
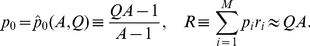
(3)We can also use 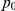 and 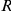 of Eq. (3) to derive Eq. (2) from the condition 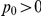 or 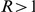. In Ref. [22], we have derived Eq. (2) as a special case of the Eigen model with a general fitness function and degradation rates.

The error rate 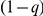 has a value between 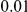 and 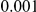 [3], [4], restricting the length of early information carriers to some number between 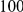 and 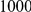 for 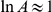, which is much smaller than 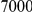 or 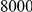 for the most primitive genome, as estimated by Kun *et al* [6], mentioned above. This is the famous error catastrophe paradox.

One hope of increasing the information content has been connected with the idea of neutral network-like fitness landscapes [6], [17]. However, the quantitative impact of this phenomenon has not been rigorously investigated. Truncated fitness landscapes have been discussed in [10] with regard to Muller's ratchet.

Summers and Litwin [11] investigated the extreme form of truncated fitness with only one Hamming class for viable mutants, and claimed the absence of an error-threshold relation in virus populations. In this paper, we use methods developed in [22] to solve analytically the model with lethal mutations and truncated fitness, and find that lethal mutants and truncated selection together can solve the paradox of the origin of life.

## Results

### Neutral Landscapes and Critical Chain Length

#### Neutral network

If two neighboring sequences (also called “nodes”) have almost the same reproduction rate, the mutation from one node to another barely changes the reproduction rate. Such a mutation is called neutral mutation. In a neutral network, every node of the network has almost the same reproduction rate as the master sequence and every node in the network can be connected to the master sequence via a series of neutral mutations. In the neutral network, the number of neighboring nodes of a given node 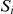 is called the connectivity or degree of that node and is denoted by 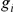. The probability of 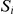 appearing is 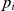. The total probability 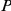 of the neutral sequences is 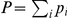, where the summation is over all nodes in the neutral network. The mean degree is given by 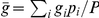. Now we have the fitness 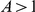 on the neutral network and fitness 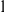 outside the network.

In the infinite genome length limit (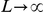) the principal term for the mean fitness of the neutral networks and the total probability 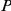 of the neutral sequences are expected to be very close to those for the single peak fitness given by Eq. (3), hence
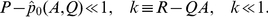
(4)Here, 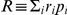 is the mean fitness computed over all sequences. The error threshold is defined from the condition 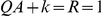.

We consider the case that in the network there is a node with a maximal degree (also called the “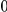-th sequence”), for which the degree 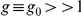, and other nodes have small degree, 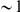. Having a large parameter 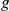, we can solve the evolution problem in the neutral network within 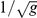 accuracy. We assume, and our calculations confirm this conjecture, that the impact of neutrality on the mean fitness must be defined by the largest degree of the neutral network. We denote by 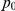 the probability of having a sequence with 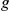 neutral neighbors, and by 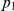 the probability of any of these 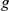 neutral sequences. Consider the steady state solution of Eq. (1). With the accuracy 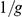, we obtain the system of equations for 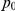 and 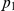:
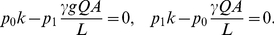
(5)In the first equation in Eqs. (5), we omit the contribution from 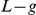 non-neutral neighbors. In the second equation, we omit the contribution from the second Hamming class. Both these corrections are proportional to 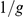. Using the balance condition 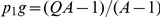, we solve Eq. (5) to obtain
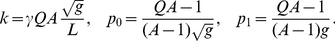
(6)Thus, 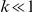, which is consistent with Eq. (4).

The above calculations illustrate well that the impact of neutrality is determined by the maximal degree 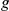, and its effect on the critical chain length is only of the order 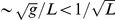 because 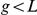. We can also derive the probability for a sequence with larger Hamming distances from 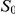. For this purpose, let us assume now that a neutral mutant from the first class has a neutral neighbor from the second class with a relative probability 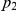. It follows from Eq. (1) that 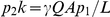 or 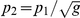, which gives
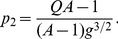
(7)Repeating the derivations for the probability of a neutral sequence at Hamming distance 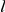 (along the neutral network) 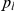, 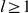, we can show that the probability of having neutral sequences at the Hamming distance 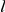 along the neutral network from the master sequence is:
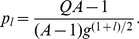
(8)


**Figure 3:**
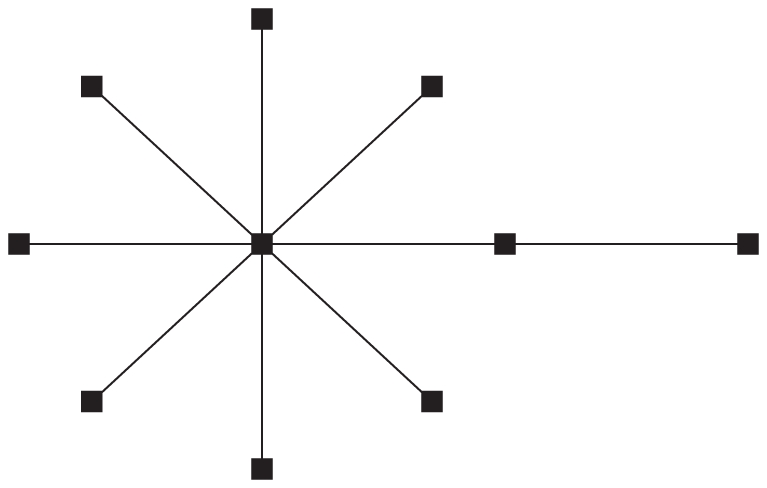
Neutral network-like fitness landscapes. There is a sequence with 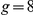 neutral neighbors and a tail of neutrals with the length 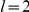.

**Figure 4:**
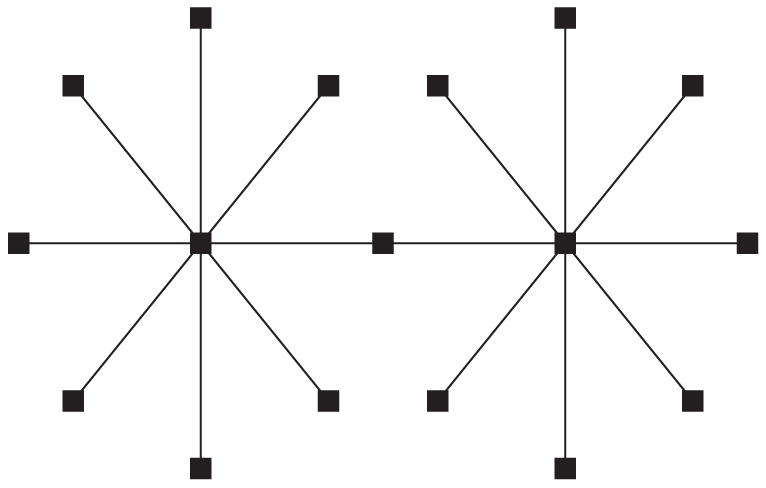
Neutral network-like fitness landscapes. There are two sequences with 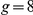 neutral neighbors, with 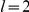 distance between two centers.

We can use results by Nimwegen *et al* [14] to check the reliability of our result. For a very small 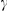, Eqs. (4) and (6) in [14] by Nimwegen *et al* can be written as
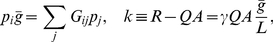
(9)where 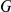 is the adjacency matrix of the neutral network: 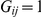 if types 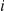 and 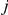 are neighbors, and otherwise 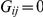. In the case of the Eigen model, considered in the current article, Eq. (9) is derived for the finite 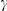 as well. Equation (9) could easily be solved for the types of neutral network shown in Figs. 3 and 4.

For the neutral network type of Fig. 3, we obtain
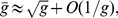
(10)of which the derivation is presented as Case 5 in **Materials and Methods**. Equations (9) and (10) are consistent with Eq. (6).

For the neutral network type of Fig. 4 with a large degree 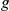, we have
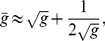
(11)of which the derivation is presented as Case 6 in **Materials and Methods**. Thus our results are consistent with those obtained by Nimwegen *et al* [14].

As another test of Eq. (6), we present the derivation for the simplest case of one central sequence and its 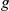 neutral neighbors 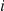, 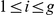, as follows. Nonzero matrix elements of 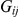 are 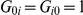. Due to the symmetry, Eq. (9) transforms into the system of equations: 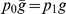, and 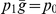. Multiplying the first equation by the second gives 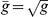. With such a result in the second expression of Eq. (9), we can obtain the first expression of Eq. (6). Thus our results are consistent with those obtained by Nimwegen *et al* [14].

#### Mesa-type fitness landscape

The mean fitness of the *mesa* landscape has been calculated first in [21]–[23], then later analyzed in [28]. In a typical case, the high fitness extends to the Hamming distance of 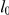, i.e., 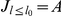 and 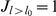, where 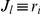 is the fitness at Hamming distance 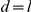 from the wild sequence 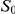, see e.g., Eq. (13) in [22] with 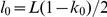 and 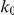 is defined in [22]. We derive rigorous solutions for several cases in “Correction terms for meta-type fitness landscapes” in **Materials and Methods**.

Solutions for various fitness landscapes are presented in Table 1. We note that because of the neutrality, there is a slight increase in the values of the mean fitness: 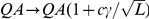, where 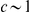. Results for Case 3 and Case 4 in Table 1 show how by removing a single point, the 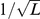 correction term changes. This sensitivity of the mutant spectrum is quite typical. These results were derived by the 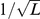 expansion. Higher-order correction terms can be derived as well, i.e., the accuracy of the results included in Table 1 can be controlled.

**Table 1:** Correction terms 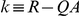 (in the third row) for 4 different cases of mesa-type fitness, where 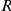 is the mean fitness and 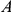 is the fitness at the wild sequence 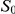.

case	1	2	3	4
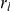	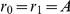	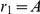	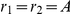	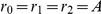
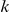	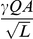	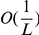	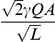	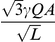

1. 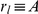 is the fitness at the Hamming distance 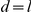 from 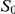.

2. 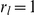 for all 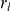 that are not explicitly included in the table.

Table 1 implies that the change in mean fitness and error threshold (defined by equation 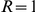) due to neutrality are rather small, of the order 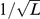.

The expression of Eq. (5) in [22] can be considered as the mean fitness 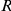 of the model defined by Eq. (1) in [22]. We have used Eq. (5) in [22] to discuss the effect of the flat fitness function, defined by Eq. (13) in [22]. It is easy to show that the modification of 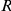 is of the order 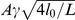 for 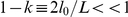. The 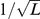 result derived first in [22], was observed later in [28].

#### Neutral thick hierarchic tree-like fitness landscape with decreasing thickness of branches

The third scenario of neutrality is connected with a neutral thick hierarchic tree fitness landscape. It is a solvable model for the fitness landscape, where the genome is fractured into several parts with regard to the neutral property. For the Hamming distance 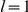 from the peak configuration 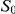 with fitness 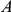, there are 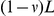 sequences with fitness 1 and 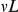 sequences with fitness 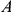. At the Hamming distance 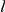 there are 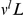 neutral mutants. Such classification is continued until the Hamming distance 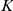 from the peak configuration 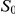. The sequence with a Hamming distance larger than 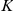 always has the fitness 1. In this model, the fraction of neutral mutations decreases with the Hamming distance exponentially until the maximal Hamming distance 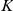.

We derive a close system of exact algebraic equations for any finite 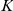, then check the quick convergence of the mean fitness shift with the 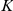. We assume that such a model is close to the reality. In “Corrections for neutral landscape with thick hierarchic tree” in **Materials and Methods**, we calculated the corrections to the mean fitness. We find for the mean fitness the correction factor to be only of the order 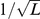.

Following the results of [8], [30] for one point neutral mutants we take the value 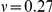; in “Corrections for neutral landscape with thick hierarchic tree” in **Materials and Methods** we further take 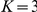 for our thick tree model to obtain
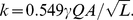
(12)The thick neutral-network fitness landscapes considered here change the error threshold only by a few percent, and certainly cannot solve the error-catastrophe paradox of the origin of life.

#### Extensive neutrality

Let us discuss different versions of extensive neutrality to clarify limits of applications for our non-extensive neutrality formulas. The main question we should address is, in which situations will the extensive neutrality change our result about the 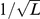 factor? First of all, we consider several situations with “thick” neutrality sub-manifolds (parts of neutral manifolds, connected with each other via neutral pathways), including partially mesa landscape, thick sub-manifold with a long thin tail, two thick sub-manifolds connected by a thin path, and two overlapping mesa landscapes.

In the section “Neutral selective Value” of [22], we have considered the “Partially mesa landscape”. Consider some fraction 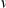 of alleles. Any mutations of these alleles with the total number 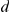 or less is neutral. We define such a landscape as a “partially mesa landscape”.

For the increase of mean fitness due to neutrality in such landscapes, the left-hand side of Eq. [19] in [22] gives:
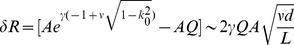
(13)where we consider the case 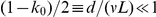 and denote 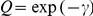. If the fitness does not depend on the values of nucleotides in some part of a genome with the length 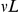, then the error threshold changes 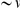 because the effective genome length becomes shorter, 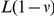. In such a case of a partial mesa landscape (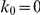 in Eq. (13)), there is no small factor 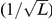, the case considered in [6], [16], [17]). Equation (13) coincides with Eq.(4) in [17] for 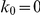.

It has been observed in experiments that there are rather long neutral pathways in a sequence space. Figure 3 is a schematic diagram of a landscape with long neutral paths in sequence space. But such long paths cannot significantly change the mean fitness, as has been well illustrated by Eq. (10) of the present paper. The long tail contribution to mean fitness is negligible compared with the "thick" part of the neutral manifold (supposed to be a partially mesa landscape), and the latter gives an increase of mean fitness 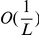.

If we have several “thick” parts of a neutral manifold, connected together by thin neutral paths, the common increase of mean fitness due to neutrality is just equal to the increase by one with the maximal "thickness". Figure 4 is a schematic diagram for such a landscape with two “thick” parts of neutral manifold connected together with a thin neutral path. Equation (11) illustrates this phenomenon in the case of two identical "thick" neutral sub-manifolds, connected with the thin neutral path.

Let us consider overlapping mesa landscapes. In the simplest case, we have two reference sequences at the Hamming distance 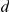, and the sequences are neutral until 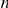 mutations from either of the reference sequences.

We performed numerics for the parallel model [18], which is closely connected with the Eigen model [20]. In Table 2, we provide the results of the mean fitness for different distances between central sequences. We see only a slight increase in the neutrality impact 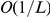, and the maximal fitness increase appears at a small distance 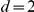. While we have done numerics only for two overlapping mesa landscapes, it is reasonable to assume that the same 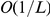 scale of corrections should still be valid in cases with several overlapping mesa landscapes.

**Table 2:** Numerically obtained mean fitness 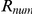 for the parallel model with two overlapping mesa landscapes around two sequences, with Hamming distance 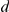 between central sequences, and with the maximal neutral mutation number 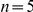.

d	0	1	2	3	4	5
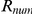	1.033287	1.033386	1.033395	1.033288	1.033287	1.033287

The results listed in Table 2 are for the parallel model with 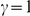 mutation rate per genome and 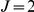 difference between fitnesses of the sequences on the neutral manifold and the reminder sequences.

#### Experimental data analysis for the effect of extensive neutrality

Until now we have assumed a perfect neutrality, when the neutral mutants have exactly the same fitness as the master sequence 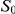. Let us now analyze the experimental data of [8], [30], to clarify the possible modification of our theoretical conclusions for imperfect neutrality, corresponding to observed data. The authors of [8], [30] defined as “neutral” sequences having 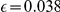 less relative fitness than the master sequence has. How large is such a decrease in fitness? Our formulas for the neutral network are valid when the nearest neighbors have a decrease in relative fitness 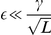. Otherwise, when 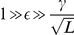, Eq. (5) gives another result for the change in mean fitness, when we have a central sequence with fitness 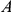 and its 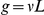 neighbors with fitness 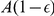:
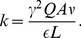
(14)


Consider now the case of extensive neutrality: we assume that the multiple neutral mutations act independently, and thus the relative Wrightian fitness after 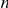 neutral mutations is [31]



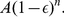
(15)


Using Eq. (5) from [22], we again obtain Eq. (14). Thus the nearest neighboring neutral mutants make the bulk of the contribution to the increase in mean fitness due to neutrality, according to the data by [8], [30].

All our formulas are for the selective phase where 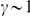. Putting 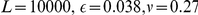, we find that the error threshold is changed by only 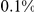. This result does not change even if we take into account the epistasis, observed in [30].

### Lethal Mutants

The existence of lethal mutants is well established experimentally [8] and there have been several approximate results [32], [33]. A rigorous investigation of the phenomenon started only recently. In [9], we calculated the exact mean fitness for the model with a general symmetric fitness landscape and lethal mutations, including the case of the single peak landscape as a special case. The exact error threshold for the latter case was derived by Tejero *et al.* [35], who also used approximate methods and ideas of [36] to study the extinction threshold.

The extinction phenomena in bacteria originate from “internal” degradation: a mother bacterium is replaced by two daughter bacteria (with possible mutations), therefore *the mother disappears* after the self-replication cycle. In contrast to the case of bacteria, we assume that the self-replicating RNA molecule does not disappear after providing copies, and therefore can participate in self-replicating events multiple times. Therefore, there is neither an “internal” degradation process nor connected extinction threshold phenomenon in our case; see the subsection **Extinction threshold** in **Materials and Methods**. Here we will calculate the probability distribution for a single peak fitness model with lethal mutations, which was not done in [34] and [35].

Let us consider a single-peak fitness model, in which lethal mutants are randomly distributed in the sequence space. First of all we define accurately the distribution of lethal mutants (zero fitness) in the sequence space. The number 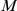 of non-lethal sequences scales as some degree of the total sequence number,
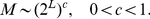
(16)How can we dilute the sequence space by lethal sequences? Let us choose a reference sequence 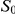 (the sequence with a high fitness 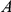 in the case of a single peak fitness model). In the first Hamming class with Hamming distance 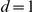 from 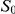, we have 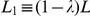 non-lethal mutations and 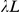 lethal mutations; in the second class with 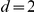, there are 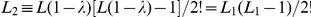 non-lethal sequences;…; in the 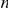-th class with 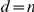, there are 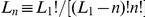 non-lethal sequences. Thus the total number of non-lethal sequences is
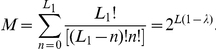



Comparing this 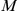 with that of Eq. (16), we have 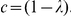 In the above derivation, we assume that any sequence having several mutations, including at least one lethal mutation from the one-point lethal mutation list is also lethal, and we ignore the combinations of deleterious mutations (synthetic lethal). Such a picture is quite realistic for RNA viruses [8], [30].

For the lethal mutants with parameter 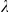, in the subsection **Lethal mutants** in **Materials and Methods**, we derive with 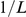 accuracy
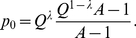
(17)In the infinite population limit, the error threshold can be determined by the condition
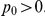



Thus from 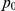 in Eq. (3), one can obtain the error threshold in Eq. (2) derived by Eigen. In the infinite population limit, 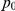 in Eq. (17) implies that the error threshold for the case with fraction 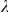 of lethal mutants is given by
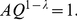
(18)


Current experiments suggest that the probability 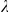 of one point lethal mutants is 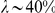, i.e., about twice as high as the probability of hitting the neutral mutants [6], [8]. For 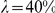, the error threshold constraint is relaxed by a factor of 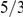. This is insufficient to solve the error threshold paradox. It could be solved by increasing the degree of lethality 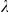 and involving the truncated selection (see next section). Equation (18) shows that while the lethal mutations change the error threshold, the fraction of the master sequence decreases with the high mutation rates (small 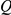). If there is an extinction threshold in the population (the population disappears below a minimal value of the mean fitness) [35]–[37], then even the lethal mutations cannot rescue the situation: the selective phase disappears.

The existence of the error threshold is a fundamental phenomenon, connected with the Shannon optimal codes in information theory [38], while the extinction threshold is a case dependent, non-universal phenomenon.

### Truncated selection with lethal mutations

Consider a fitness landscape with “truncated selection” (Fig. 2) [10]–[12]. We take 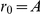; 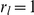, 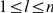; and, 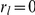 for 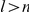, where 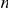 is the truncation parameter. We denote by 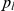 the probability of having a sequence from the 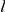-th class. In [12], we solve analytically the truncated fitness landscape for the case of large 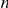, and perform numerics for the finite 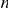 case. For the large 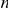, we find in [12] that the error threshold transition is fractured into two separate transitions.

Now we will derive analytical expressions of the mean fitness, and consider the case of truncated mutation with lethal mutants in the case of small 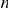. For the master type, we have 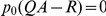, where 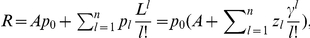 where 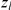 are defined by Eq. (54) in **Materials and Methods**. Then we define the 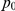 by the equation
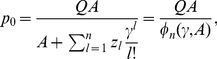
(19)where the function 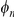 is defined in Eq. (55) in **Materials and Methods**. For the truncated selection in the presence of lethal mutants, Eq. (19) should be changed to:
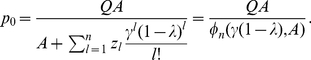
(20)


We put the error threshold condition within the 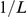 accuracy:
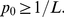
(21)Were a population size 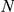 available from experiments, we could, instead of Eq. (21), use another constraint for the 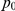:
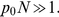
(22)The justification for the conditions given in Eq. (22) is that molecular population size 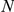 should be high enough to produce deterministic features. The population size should be large enough to avoid the loss of the master sequence due to the Muller's ratchet effect. In reality, it is not easy to obtain the value of 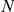. Thus in the following, we will use Eq. (21) to estimate the critical length.

Having 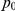 given by Eqs. (17) or (20) for several typical parameters of the models, our estimates of the critical length with Eq. (21) are gathered in Table 3. The results for the neutral network case and neutral thick network case were obtained with the use of Eq. (6) with 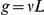, and Eq. (12), respectively. The degree of neutral mutations is taken as 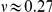 [8]. We have verified that for the four-letter alphabet the impact of neutrality is smaller by a factor of 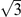, but other entries in Table 3 remain unchanged.

**Table 3:** The maximal allowed genome length 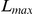 obtained from different conditions (equations) for several values of the parameter of truncated selection 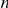 and the degree of lethal mutations 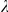 when 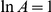 and 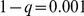.

Conditions	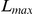	n	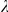
Eq. (2)	1000		
Eqs. (3), (21)	998		
Eq. (6) with 27%-neutrality	1016		
Eq. (12) with 27%-neutrality	1017		
Eq. (18)	1666		0.4
Eqs. (17),(21)	1664		0.4
Eqs. (17),(21)	2000		0.5
Eqs. (17),(21)	4915		0.8
Eq. (18)	5000		0.8
Eqs. (17),(21)	6300		0.85
Eq. (18)	6666		0.85
Eqs. (17),(21)	7800		0.9
Eq. (18)	10000		0.9
Eqs. (20),(21)	4650	4	0.4
	5430	3	0.4
	6500	2	0.4
	5050	4	0.5
	5800	3	0.5
	6750	2	0.5
	7050	4	0.8
	7450	3	0.8
	7900	2	0.8
	8200	3	0.9
	8310	2	0.9

If we assume 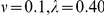, and extensive neutrality according to Eq. (15), then the results of Table 3 are changed slightly by 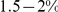.

## Discussion

In this work we have rigorously investigated the error-threshold problem for evolution with neutral and lethal mutants, and with truncated selection. We have calculated the change in mean fitness (e.g. 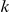 in Eqs. (4) and (6)) due to neutrality for the neutral network with a high degree 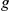 at some node. We also considered the neutrality phenomenon for a more involved case, in which the fraction of neutral mutants among all multiple mutants decreases exponentially with the Hamming distance from the master sequence. Then we found that the neutrality changes the mean fitness and the error threshold by only a few percent 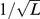 and certainly cannot solve Eigen's error threshold paradox. The formulas considered correspond to perfect neutrality, where neutral mutants have exactly the same fitness as the master sequence. We also considered the case of imperfect neutrality: the neutrality case according to the data of [8], in which there is a small decrease in fitness after mutations. Assuming extensive neutrality in such a case (multiplicative character of fitness for those mutations), we find that such a neutrality alone can change the error threshold by 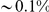. Thus neutral mutation alone cannot solve the error threshold paradox. Our result is rather general, as, according to experimental data [8], an assumed extensive neutrality gives the same increase in mean fitness as the model with only the nearest neighboring neutrals.

We solved exactly the model with lethal mutations. Both phenomena, the occurrence of significant proportions of the neutral and the lethal types, suppress the error threshold in a similar way, while there is neither a small factor 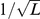, nor a fine-tuning problem in the case of lethal mutations. The effect of the lethal mutants is, however, easier to realize than the effect of neutrality, even after a billion-year evolution. The difference in the impacts of these two mechanisms is thought to have been even more pronounced at the origin of life. We have provided evidence that in modern RNA, the presence of lethal mutants can cause an increase in the error threshold by as much as 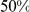.

We have showed that the Eigen's error threshold for the origin of life can be relaxed, provided the presence of the lethal mutants is aided by truncated selection (see Table 3). For example, in the case of RNA molecules, the maximum length of chains is considerably extended when lethal mutants with an 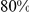 lethal probability are included in the model, together with truncated selection. In the absence of truncated selection the probability of the master-type sequence would be negligible, which in turn would require enormously large molecular populations for reactions to happen. For maintaining a continuous replication, it is important to have in the population both lethal mutants and viable mutants. Moreover, the latter should be restricted to 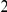 to 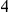 base exchanges. If we assume 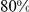 lethal probability and 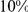 neutrality, then the neutrality can change the error threshold by 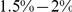.

One of the questions of interest concerns the organization of truncated selection in pre-biotic evolution. Only recently it has been realized that proteins are not random heteropolymers but their sequences are formed following a tentative design (for a review see [39], [40]). Developed during evolution, this design entails, for example, the robustness of the genome against mutations. In the context of applications to RNA, the concept of design was recently studied by Zorn *et al* [41]. It is reasonable to assume that the degree of design and the robustness were poor at the beginning of evolution. For example, initial evolution might have followed a scenario in which the truncated selection took place in a population with a large number of lethal sequences. As shown here, in this example under poor organization, the error catastrophe could have been avoided.

The key point of our study is that no matter where the beginning of life was (it is an obligatory property of the matter, as has been assumed in [42]), if it was through autocatalytic reactions, it had to be accompanied by lethal mutants with truncated selection.

In summary, in populations that contain about 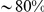 of lethal mutants and provide for the simultaneous truncated selection with the truncation parameter 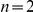 or 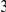, the primordial genome can reach the critical length of 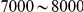 estimated by Kun, *et al.* [6], and Eigen's error catastrophe can thus be solved.

There are three essential stages in the origin of life [42]. The First is the preliminary stage, with the preparation of the proper bio-molecules for the starting point [42]–[44]. The Second is connected with self-replication of macro-molecules [42]. The Third gives the protocells [45]. The present paper studies the second stage. The error threshold problem exists for both the second and third stages. In the second stage there is a replication of molecules using a template, while in the third stage the mother protocell divides into two protocells. The mechanism we suggested solves the error threshold for the second stage, but not for the third stage: too much lethal mutation push the population to the extinction threshold [15] and the self-replication of proto-cell will stop. Thus a protocell should have auto-proof mechanism of self-replication to suppress the mutation rates.

Let us briefly discuss our results in view of alternative ideas to solve the error threshold paradox. All of our derivations and conclusions correspond to the case of replication of a pre-biotic molecule using a template. An alternative mechanism to avoid the error threshold could be connected with the self-replication of the network of molecules [46], [47], in which several enzymes catalyze the generation of each other. Such a mechanism increases the value of the joint fitness of the "peak" configuration of a couple of sequences, which is useful in avoiding the error catastrophe. For the origin of life, we need some minimal pool of genes, which could be provided by two molecules (replicating together) with a shorter length for each chain. Unfortunately, the information contents of the two sequences in [47] are almost identical, therefore such a concrete mechanism could not provide a larger number of genes than the single sequence. In the case of a connected replication of several RNA-like molecules with different information content and self-sustaining amplification of the whole molecular group, such a mechanism, combined with the lethal mutations, can easily solve the error paradox, and the mathematical tools developed in the current article could be applied in this case as well.

Peck and Waxman [48] proposed the evolution model with recombination and concluded that the truncated selection and recombination could solve the error paradox. We agree with the importance of the truncated selection, while have not see serious argumentation for the importance of recombination to solve the error threshold catastrophe. They used non-zero degradation in their model, while have forgotten to analyze the extinction threshold. A rigorous consideration of the single peak fitness landscape with the simplest version of recombination in [49] proves that the recombination does not change the (mean fitness) error threshold for the long genome and hence could not solve the error paradox. For the short genome the recombination even slightly suppresses the selection (the mean fitness decreases) for the single peak fitness case [50].

Rajamani, *et al.* [51] have considered the mechanism of self-replication cycle in details, assuming slow reaction rates for the mutants due to “mismatch stalling”, which can somehow change the error threshold, when the error probability per nucleotide times the “stalling” coefficient [51] is larger than the fitness ratio (wild sequence fitness to the other non-lethal sequence fitness). Actually the considered phenomenon is equivalent to some increasing of the fitness ratio 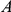. The phenomenon depends on the concrete details of the self-replication cycle. One should consider this phenomenon together with lethal mutations, truncated selection and finite period of generation [52].

Our work helps to solve a puzzle in the second stage of the origin of life [42]. Such result and other recent advances in models of cells [53]–[56] and minivirus [57] will provide clues for understanding the evolution from the second stage to the third stage of the origin of life.

## Materials and Methods

### Correction terms for mesa-type fitness landscapes

Consider the steady state solutions of the Eigen model [1], [2] for the fitness landscapes with two classes of sequences: with a high fitness 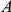 and with a lower fitness 1 (one).

#### Single peak fitness model

Consider the fitness landscape
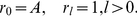
(23)This gives the following expression for the 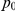 and mean fitness [22]

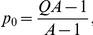


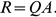
(24)


#### General case

Consider the fitness landscape where there is a high fitness 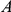 for 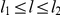 and fitness 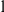 for other sequences. There is an exact equation for the mean fitness
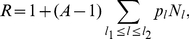
(25)where 
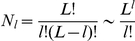
 for small 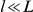. The total probability of neutral sequences is approximately the same as in the single peak fitness model,
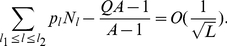
(26)


We assume the following ansatz for 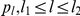


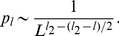
(27)The mean fitness 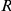 is almost the same as for SP case:
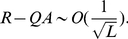
(28)The majority of population is in the highest Hamming class and we have
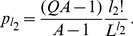
(29)Then from Eq. (1), we have the following system of equations
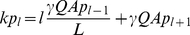
(30)where 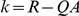. For 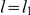 we miss the first term 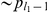 and for the 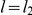 we missed the second term 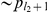 in Eq. (30). The higher terms 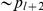 as well as the lower one 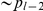 are missed, because their contribution are suppressed due to a small factor 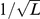.

#### Case 1. The simple mesa

Consider now the case, when besides the 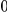-th configuration there is a high fitness at the Hamming distance 1
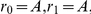


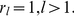
(31)From Eq. (29), we have 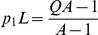. Equation (30) implies that
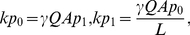
(32)where 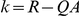. We have a solution:
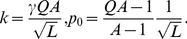
(33)


#### Case 2. Mesa with the hole

Consider now the case, when there is high fitness at the first Hamming class
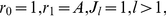
(34)We have in the bulk approximation
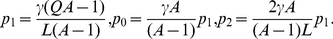
(35)As 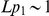, then 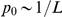, therefore there are no 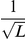 corrections now, just 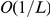 ones. To get 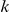 we consider the equation for 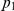 with the small corrections:
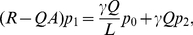
(36)which gives
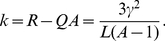
(37)


#### Case 3. Band with zero at the center

Consider now the fitness landscape
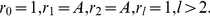
(38)Now we have
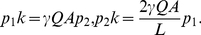
(39)We derive immediately
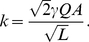
(40)


#### Case 4. Thick band

Consider now the case, when
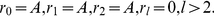
(41)We have equations
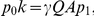


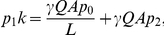


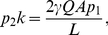
(42)which imply
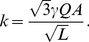
(43)The results of Cases 1–4 are listed in Table I.

#### Case 5. Model with fitness by Fig. 3

Denote the number of all one point neutral mutants by 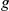, 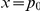, and by 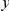 the probabilities of 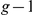 similar neutral neighbors (without neutral tails), by 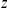 the probability for non-symmetrically located one point mutation neighbor of the master sequence, and by 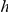 the probability of two point mutation neutral mutant. We have a system of equations for variables 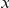, 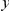, 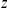, 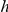 and the average number of degree for the whole neutral network 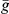.
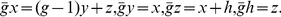
(44)Putting 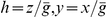, we derive an equation for 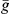:
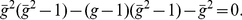
(45)For the large 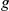 we have
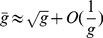
(46)If we take the longer tail, 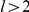 in the Fig 3, only 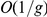 terms are affected in Eq.(46).

#### Case 6. Model with fitness by Fig. 4

Denote again by 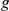 the total number of neutral neighbors of two nodes with the largest degree, 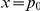, and by 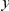 the probabilities of 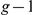 similar neutral neighbors, by 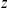 the probability for non-symmetrically located one point mutation neighbor of the master sequence. We have a system of equations for 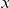, 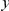, 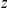, and 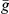


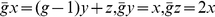
(47)Putting 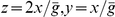, we derive an equation for 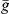:
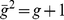
(48)For the large 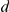 we have
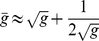
(49)We took the Hamming distance 2 between two centers of thick sub-manifolds. If we take more Hamming distance, then the second term in the last equation should be 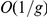.

### Corrections for neutral landscape with thick hierarchic tree

Here we follow the terminology of Sec. I to call a nucleotide as a spin. In the model of neutral landscape with thick hierarchic tree, the 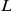 spins are partitioned into 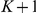 different groups, with 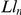 spins in the 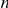-th group, where 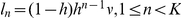, and 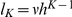. Thus the maximal distance on the tree from the reference sequence equals 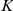. There are at most 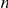 neutral point-mutations in the 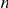-th group. We take 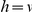. Compared with the neutral network of the previous subsection, now the hierarchic tree has a thick tube instead of thin lines. What we are doing is equivalent to solving Eq. (9) using the symmetry: collecting the same type of sequences together. We should distinguish among different probabilities for the sequences that are obtained from the central sequence after mutations of the spins from different groups. We denote as 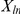 the probability of having a sequence from the 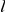-th Hamming class but with the spins from the 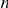-th group. Such sequence has 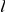 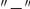 spins and 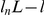 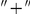 spins from the same group of spins. From the Eigen model equations we get:
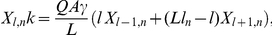


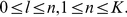
(50)and we identify 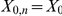 and 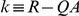, also put 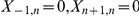.

We have a complete system of equations to define 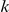 and 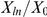. In the case when 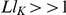, we can further simplify the system of equations,
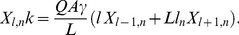
(51)


Consider first the case of 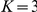. We have 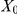 for the 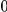-th class probability. For the lowest group we have 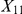 for a sequence in the first Hamming class, i.e., when the distance from the master type is 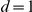. For the second group of sequences, i.e., for those obtained via mutations of the second spin group, we have 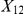 in the first Hamming class, and 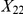 in the second Hamming class. For the third group of sequences, we have 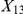 in the first Hamming class, and 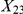 in the second Hamming class, and 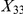 in the third class. We derive the following system of equations:
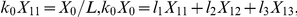


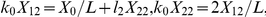


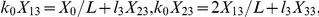


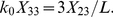
(52)


Here 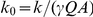 is an eigenvalue of a matrix. The second equation in the first line of Eq. (52) was derived directly from Eq. (1).

After re-scaling 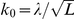, 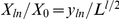 we have a system of equations without large parameter 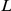:
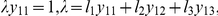


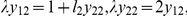


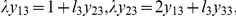


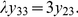
(53)In the last equation 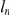 are functions of 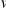. For 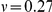 we have a correction to the mean fitness 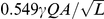. The 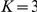 gives the results of large 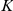 with the accuracy 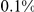.

### Lethal mutants

We will calculate mean fitness with 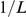 accuracy. Let us first consider the case without lethal mutants. We denote by 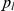 the probability of having a sequence from the 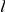-th class, and by 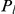 the probability of having the 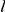-th class. In the 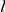-th Hamming class there are 
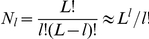
 sequences and 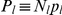. Let us denote 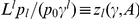, and therefore 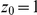. The recurrence relations for 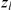 are [22]:
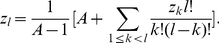
(54)Having the values of 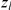 we can calculate 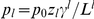.

We have for the mean fitness 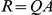. On the other hand, mean fitness is defined as 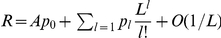. Thus with the 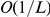 accuracy,
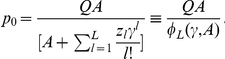



Using the master degree probability 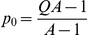, we get,
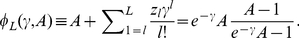
(55)


Consider now the case with lethal mutants. In the 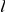-th Hamming class we have 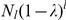 non-lethal sequences: each of which has the probability 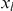, and 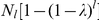 lethal sequences. For the 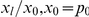 we have the same system of recurrent equations as those for 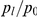 in case without lethals, therefore we can calculate 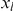 as 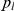. A single modification, we should replace 
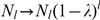
 while calculating the mean fitness expression:
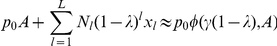


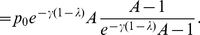
(56)For the distribution of lethal sequences with the faction 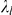 in the 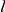-th class [33], the last equation is modified: 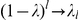.

Equation (56) defines the mean fitness. For the master type we have 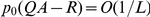. Thus we have again for the mean fitness 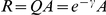. Therefore, we have an equation for the 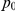


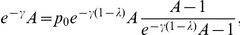
which implies Eq. (17).

### Lethal and neutral mutants

Consider a single peak sequence with a fitness 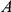, a part of genome 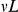 with a fitness by Eq. (15), and 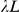 positions in genome for lethal mutations. In the selective phase we get, following to Eq.(14), a mean fitness



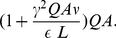
(57)Using Eq. (18) and the expression 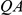 for the mean fitness of selective phase, we get the following expression for the mean fitness of non-selective phase:
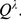
(58)Comparing the latter two expressions, we get for the error threshold:



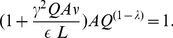
(59)


### Extinction threshold

The growth of bacterial population is through the cell division. At time 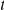, we have 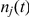 bacteria of the given type. Let us consider the division of the bacteria of type 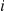 into two daughter bacteria with types 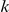 and 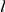 [37]. After the bacteria division the number of bacteria of the type 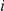 decreases for 1, and with probabilities 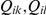 increases the numbers of bacteria with the types 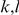.
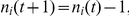


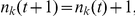


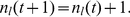
(60)We can model such a situation with the continuous time model
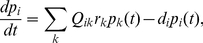
(61)where 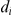 is the degradation term introduced in [2], and following to [36] and [37]. All the terms 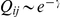. The origin of the 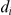 is just the disappearance of the mother bacteria after the division ("internal" degradation).

Carefully analyzing Eq. (61), the authors of [36], [37] deduced the extinction threshold, a phenomenon when the total population size decreases. There is a strict constraint
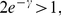
(62)otherwise the population disappears.

The origin of their conclusion is the existence of nonzero degradation rate 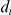, initiated by the first equation in Eq.(60).

The point is that in the case of RNA replication considered in this paper, we have another situation:
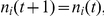


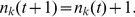
(63)


Thus while considering the corresponding continuous time model, we don't need to add the negative term, and get just the Eigen model for growing population
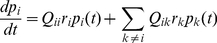
(64)Of course, it is possible some degradation due to interaction with external environment, but there is no strict constraint like the one described in Eq. (62).
